# Adaptive Management Based on the Habitat Change of *Cibotium barometz* Under Synergistic Impact of Climate and Land Use Change—A Case Study of Guangxi, China

**DOI:** 10.1002/ece3.71040

**Published:** 2025-03-13

**Authors:** Bin Feng, Yunyun Zhang, Yunfeng Huang, Huabing Dai, Chao Yang, Chengling Yang, Kedao Lai

**Affiliations:** ^1^ Guangxi Institute of Chinese Medicine & Pharmaceutical Science/Guangxi Key Laboratory of Chinese Medicine Quality Standard Research Nanning Guangxi China; ^2^ Guangxi Forest Inventory & Planning Institute Nanning Guangxi China

**Keywords:** Cibotium barometz, climate change, land use change, suitable distribution area, suitable habitat

## Abstract

With the rapidly growing demand for medicinal plants globally, the wild medicinal plant population is experiencing a sharp decline. Climate and land use change are two significant forces affecting biodiversity. Climate change impact assessment without changes in land use should mischaracterize medicinal plants' vulnerability and spatiotemporal distribution. Previous research on medicinal plants' potential distribution area by species distribution model (SDM) has focused more on their ecological suitability. However, whether the land‐use types within the suitable distribution area (SDA) meet the species' survival requirements is often overlooked. These imbalances place significant limitations upon the ability to guide anticipative conservation and sustainable utilization actions and weigh the future outcomes of different policy or management options. *Cibotium barometz* is a highly demanded medicinal plant listed as national key protected wild plant in China. For adaptive management, we assessed the suitable habitat change of *C. barometz* in Guangxi under the synergistic impact of climate and land use change by maximum entropy (MaxEnt) and patch‐generating land use simulation (PLUS) models between 2020 and 2040 under three Shared socio‐economic pathways and proposed adaptive management countermeasure. Results indicate that climate change accelerates the loss of *C. barometz* 's habitat;SDA and suitable habitat show a decreasing trend; the total area of suitable habitat is decreasing, but the suitability degree is increasing. Altitude and Precipitation of Warmest Quarter are key environmental variables for *C. barometz* distribution; SDA shows a southwest‐northeast shift, and the average elevation is rising. The areas of cropland, forest, shrub, grassland, and barren that meet *C. barometz* 's survival requirements are decreasing, and water and impervious surfaces are increasing. We propose an adaptive response to wild resource conservation based on the protected area system in southwestern Guangxi in parallel with artificial cultivation in northeastern Guangxi. The study aims to provide insights into the sustainable utilization of medicinal plants.

## Graphical abstract

*Cibotium barometz* is a highly demanded medicinal plant listed as a national key protected wild plant in China. To achieve the sustainable utilization of *C. barometz* , this study proposed the adaptive management countermeasure according to the habitat change under the synergistic impact of climate and land use changes. The study aims to provide insights into the sustainable utilization of endangered medicinal plants.

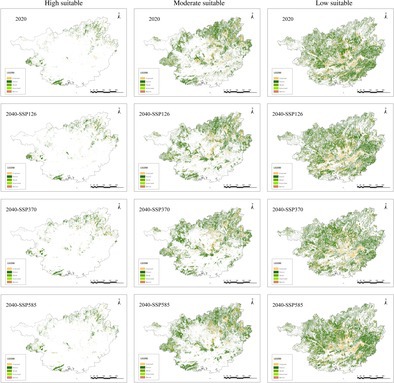

## 1 Introduction

Medicinal plants are an important national resource and play a vital role in the healthcare industry (Manish 2022). The medicinal plant market is experiencing rapid growth worldwide due to the increasing demand for herbal pharmaceuticals, natural health products, and secondary metabolites derived from medicinal plants (Wang et al. 2023). As an essential component of traditional medicine widely used in China, wild medicinal plants are in sharp decline owing to over‐harvesting, habitat loss and fragmentation, global warming, and invasion of alien species (Shan et al. 2023). The most effective method for rare, endangered, and overexploited species is artificial cultivation to provide material without further endangering the survival of valuable species (Thapa et al. 2024). An understanding of “how” and “where” conservation and artificial cultivation can be effectively implemented to address conservation‐related issues is therefore essential (Rana et al. 2020). To ensure sustainable markets and meet the future challenges of medicinal plants posed by global warming and habitat loss, a regional strategy for adaptive management is needed (Kumar et al. 2017; Zhang et al. 2023).

Climate change has an increasing influence on species distributions and populations (Northrup et al. 2019). Much research has confirmed the shift of species' distribution area under the impact of climate change (Coelho et al. 2023). The SDM is a common type of model in ecological research. It has been widely used in recent years to simulate the species' potential distribution in multiple spatiotemporal patterns (Liu et al. 2019), as well as medicinal plants (Li et al. 2020; Yang et al. 2023). Among the most widely used SDMs is the MaxEnt (Damari et al. 2020), which is applied in the assessment of suitable cultivation areas for medicinal plants (Shen et al. 2021; Zhan et al. 2022). Although climate change is emerging as a major driver of species' endangerment, currently, the most significant threat to species arises from land use change; land use change will likely continue to be a dominant driver of vulnerability into the future (Santos et al. 2021). Not only climate change but also habitat loss stressors and their synergistic effects matter in species redistribution and vulnerability (Guo et al. 2018). Climate and land‐use change may have favorable or unfavorable synergistic effects on the same species, thus amplifying their impacts, or species may respond to each threat differently, thus creating opposing effects and mitigating their respective impacts (Beissinger et al. 2023).

The development of the spatial configuration of land is contingent upon the interaction of spatial, temporal, and policy variables, the influence of which may act either to restrict or to promote the evolutionary process in question (Liu et al. 2022). The simulation of land use patch succession may serve to identify significant ecological changes and facilitate the design, planning, and assessment of ecosystem management activities (Keane et al. 2002). Cellular automata (CA) have been widely used to simulate the spatial and temporal dynamics of land use in response to natural and socioeconomic drivers, including their interrelationships at varying spatial and temporal scales (Chen et al. 2020). Many simulation models of land use change, such as PLUS (Liang et al. 2021) and future land use simulation (FLUS) (Liu et al. 2017), have been optimized based on CA depending on different theories. The development of biodiversity models and scenarios is essential for assessing the potential consequences of these threats. These approaches facilitate informed decision‐making by identifying conservation and management options that can anticipate the prospective impact of climate and land use change on biodiversity. However, there appears to be a discrepancy between the level of attention paid to climate change in scenarios exploring biodiversity and the corresponding focus on the consequences of land use change (Titeux et al. 2017). Previous studies predicting species' SDA have tended to focus more on species' potential distribution area, while the land‐use types within the SDA do not all meet the basic requirements for species survival, such as building, asphalt or concrete surfaces, and other impervious surfaces as opposed to permeable vegetation or soil surfaces (Hou et al. 2022). The two most influential factors affecting biodiversity are land use change and global warming (Ma et al. 2021). As a result, climate change impact assessments that ignore current and anticipated risks of land use change should mischaracterize the vulnerability of species (Pacifici et al. 2015). These imbalances now place significant limitations upon the ability to guide anticipative conservation actions and weigh the future outcomes of different policy or management options (Titeux et al. 2016).

Rhizoma Cibotii is a traditional Chinese medicine made from the dried rhizome of *Cibotium barometz* (L.) J. Sm., a perennial fern in the Dicksoniaceae family (Chinese Pharmacopoeia Commission 2020). It is a representative tonifying kidney drug, meanwhile widely used for osteoarthritis (Chen et al. 2022). *C. barometz* is widely distributed in Guangxi, Hunan, Sichuan, Guizhou, Yunnan, and other provinces (Yang et al. 2010; Qin 1959). Guangxi is the main production area and accounts for about 50% of China's total production (Yang et al. 2015). In 1999, *C. barometz* was listed as a national key protected wild plant, which has positively protected its wild resources. In 2018, China's General Administration of Customs removed *C. barometz* from the Catalogue of Imported Medicinal Materials. The pharmaceutical businesses in China that rely on *C. barometz* are experiencing disruption due to the prohibition of accessing domestic wild resources and importing from abroad. It is therefore essential to address the challenge of achieving sustainable utilization of *C. barometz* while protecting its wild resources.

*C. barometz* is a national key protected wild plant with high market demand. As the main production area, to address the conflict between protecting wild resources and ensuring market supply, we used a combination of MaxEnt and PLUS models to examine the synergistic impact of climate and land use change on *C. barometz* ’ habitat between 2000 and 2040 under three shared socio‐economic pathways (SSPs), assess the change of its suitable habitat, and explore the adaptive management strategy for the conservation and utilization of *C. barometz* . In doing so, we provide insights into the sustainable utilization of *C. barometz* . This study aims to offer a scientific reference for the adaptive management of *C. barometz* resources and provide insights into the conservation and sustainable utilization of endangered medicinal plants.

## 2 Materials and Methods

### 2.1 Study Area

**FIGURE 1:**
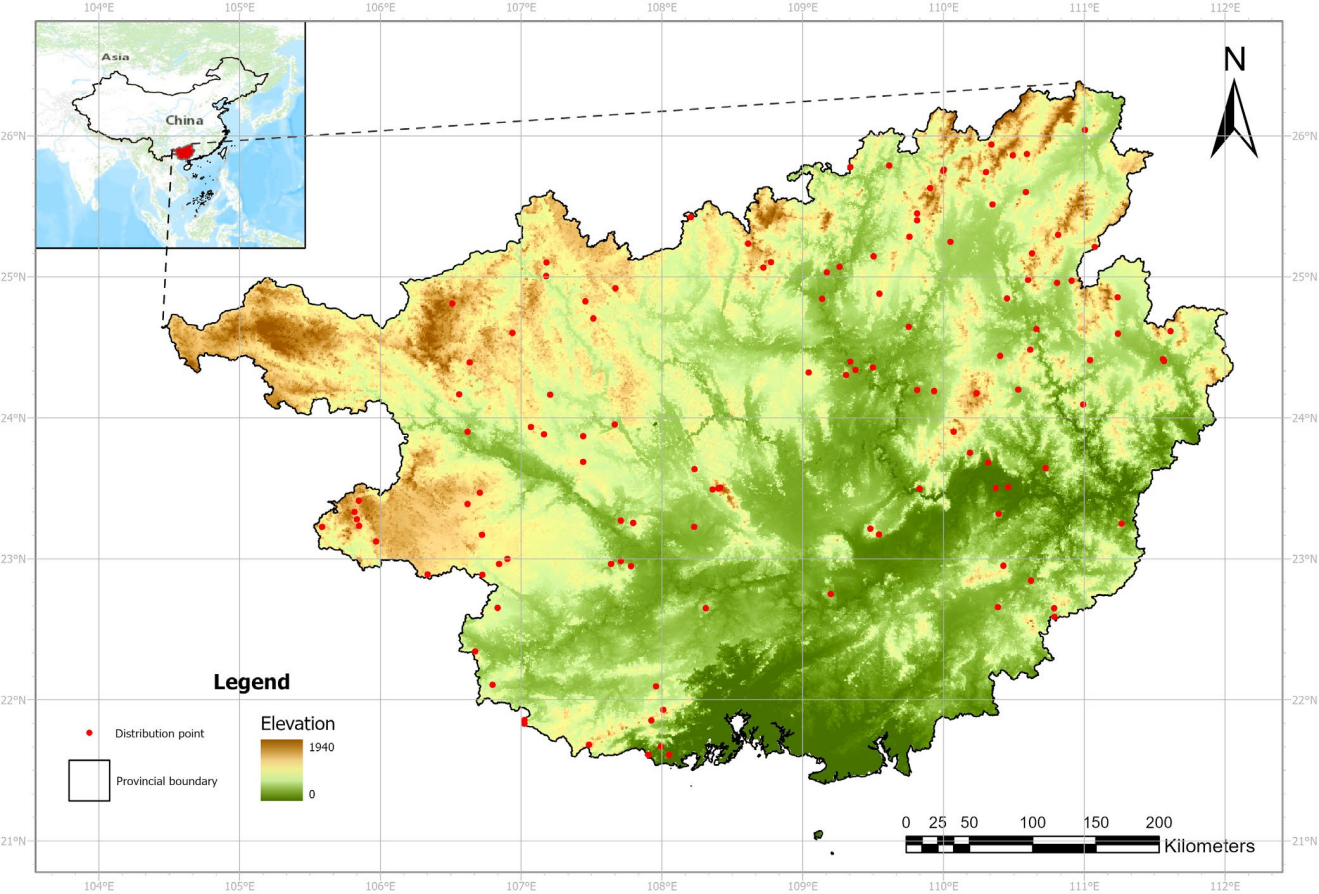
Study area and occurrences of *Cibotium barometz* .

The Guangxi Zhuang Autonomous Region (20°54′09″ N‐26°23′19″ N, 104°26′48″ E‐112°03′24″ E) is located in the south of China, with the Beibu Gulf to the south (Figure 1). It has an administrative area of 23.76 × 10^4^ km^2^, borders the Socialist Republic of Vietnam to the southwest, and has a mountainous and hilly basin landform. The topography of the region exhibits a pronounced elevation gradient, with the highest elevations situated in the northwest and the lowest elevations in the southeast. The land slopes from northwest to southeast. The climatic and altitudinal gradients in Guangxi, in combination with the diverse ecological habitats, have facilitated the growth of a rich and distinctive array of medicinal plants, which represent an invaluable source of raw materials for the traditional medicinal systems of the region. Guangxi is the main distribution area of *C. barometz* in China. According to previous research, *C. barometz* is widely distributed in Guangxi, with the main distribution area in the northeast and south of Guangxi (Chinese Pharmacopoeia Commission 2020; Yang et al. 2015).

### 2.2 Suitable Distribution Area

We employed MaxEnt to simulate *C. barometz* ’ SDA of the present and 2040 under three SSPs. MaxEnt is an ecological niche model that utilizes the maximum entropy algorithm (Phillips and Dudík 2008); it simulates species' potential distribution accurately with only species occurrence and environmental variables (Wang et al. 2020; Elith et al. 2011).

#### 2.2.1 Data Collection and Processing

The occurrence of *C. barometz* was obtained from The Fourth National Survey on Chinese Materia Medica Resources, the Global Biodiversity Information Facility, China Digital Herbarium, and literature review. To avoid overfitting caused by sampling bias, we used the spatial autocorrelation reduction tool of SDMtools 2.0 add‐on ArcGIS 10.8 to choose one occurrence from each 2 km × 2 km grid for autocorrelation reduction processing (Boria et al. 2014; Brown 2014). A total of 123 valid occurrences were selected (Figure 1).

The potential distribution area of *C. barometz* was simulated using MaxEnt with 25 environmental variables, including 19 bioclimatic variables, 3 topographic variables, and 3 soil variables (Table S1). The bioclimatic variables (1700–2000) were obtained from the WorldClim dataset version 2 to simulate the near‐current distribution of *C. barometz* (Fick and Hijmans 2017). The bioclimatic variables (2021–2040) were obtained from Worldclim dataset version 2.1 of the Coupled Model Intercomparison Project 6 (CMIP 6) to simulate the distribution of *C. barometz* in 2040. The bioclimatic variables of the ACCESS‐CM2 were chosen for their excellent simulation performance in Southwestern China (Jin et al. 2022). The bioclimatic variables were used for both current (2020) and future simulations with a spatial resolution of 30 arc sec (~1 km). The study employs bioclimatic variables from 2021 to 2040 to simulate the potential distribution of *C. barometz* in 2040, considering three SSPs that represent the most optimistic and pessimistic greenhouse gas emissions over the following few decades (Dai et al. 2021). SSP126, SSP370, and SSP585 mean sustainability scenario, regional rivalry scenario, and fossil‐fueled development scenario, respectively. Elevation, slope, and aspect were obtained from the ALOS Digital Elevation Model (DEM). Three soil variables (pH value, soil organic matter, bulk density) were obtained from the Harmonized World Soils Database version 2.0 (https://gaez.fao.org/pages/hwsd).

Raster layers preparation for MaxEnt input and mapping output was carried out using ArcGIS 10.8. All variable raster layers were projected to WGS_1984_UTM_Zone_49N and resampled to the unified resolution as bioclimatic variables, with a grid size of 500 m × 500 m. Raster layers were converted to ASC format from TIFF, with a unified boundary of Guangxi Zhuang Autonomous Region.

#### 2.2.2 MaxEnt Modeling

The modeling parameters were set as follows: 75% of occurrences were assigned to training data, while the remaining 25% were assigned to test the model (Dai et al. 2021); Jackknife was selected to test the significance of environmental variables; the Cloglog results with values ranging from 0 to 1 were chosen as the probability of species distribution (Elith et al. 2011); The replicated run type was set as Bootstrap; A total of 15 replicates were employed to generate the response curve, with the remaining parameters selected by the default settings (Phillips and Dudík 2008). The area under the receiver operating characteristic curve (area under the curve (AUC)) was used to assess the modeling performance. Threshold values for the AUC are used as an independent measure to verify the accuracy of model outputs. These values range from 0 to 1, with values closer to 1 indicating the model is more accurate. Model performance evaluation is divided into the following 5 grades: invalid (≤ 0.6), poor (0.6–0.7), satisfactory (0.7–0.8), good (0.8–0.9), and excellent (0.9–1) (Phillips and Dudík 2008). The importance of variables is estimated by percent contribution.

To improve modeling accuracy, we run MaxEnt twice (Wang and Guan 2023). After the first run, variables (percentage contribution < 1%) were removed from further modeling. Pearson analysis was used to examine the correlations of the remaining variables (percentage contribution > 1%). All remaining variables with an absolute correlation coefficient lower than 0.8 were kept, while the variables with the highest percentage contribution among those with an absolute correlation coefficient higher than 0.8 were also kept for the second run. Finally, the SDA of *C. barometz* was modeled using chosen variables with statistical and biological significance as the former setting.

Both the 2020 and 2040 SDA of *C. barometz* in Guangxi were extracted using ArcGIS 10.8 based on the results of MaxEnt modeling. SDA was divided into four levels: unsuitable (0–0.4), low suitable (0.4–0.6), moderate suitable (0.6–0.8), and high suitable (0.8–1).

### 2.3 Land Use Change

PLUS was employed to project land use change in Guangxi in 2040. PLUS combines an analysis strategy of land expansion and a Cellular Automata (CA) model using multi‐type random patch seeds. This approach could quantify the drivers of land expansion and simulate the patch growth of multiple land use types at a fine‐scale resolution, thus achieving the prediction of land use change (Liang et al. 2021).

#### 2.3.1 Data Collection and Processing

China Land Cover Dataset (CLCD) of 2000 and 2020, with a spatial resolution of 30 m, was used to simulate LULC in 2040. LULC of CLCD in Guangxi includes seven land use types: cropland, forest, shrub, grassland, barren, water, and impervious (Yang and Huang 2021). CLCD of 2000 and 2020 was obtained from ZENODO (https://doi.org/10.5281/zenodo.5816591).

Fifteen drivers and one constraint factor were chosen from natural and socio‐economic factors (Table S2). The data of Point of Interest (POI) was obtained from Open Street Map (https://www.openstreetmap.org/), and the distance from POI was processed by the Euclidean Distance tool in ArcGIS 10.8. Data on population, Gross Domestic Product (GDP), Annual Mean Temperature, Annual Precipitation, and soil type were obtained from the Resource and Environment Science and Data Center (https://www.resdc.cn/). Elevation and slope were extracted from the ALOS DEM.

ArcGIS 10.8 was used to unify the projection coordinate system of all layers, including CLCD, environmental drivers, and socio‐economic drivers, as WGS_1984_UTM_Zone_49N, with a spatial resolution of 30 m in the unified boundary of Guangxi Zhuang Autonomous Region.

#### 2.3.2 *PLUS* Modeling

The PLUS model was calibrated over the past time interval from 2000 to 2020 to evaluate the performance and accuracy of random forest classification (RFC). 5% of sample cells were selected from the spatial variable maps and the land expansion map by using a random sampling approach. These samples were employed as input data to the RFC classifier in each instance of an increase in a land use category. A total of 15 predictor variables were used for the construction of each tree split, with 50 trees employed in the generation of the final RFC models and the subsequent estimation of growth probability maps for each land‐use type (Liang et al. 2021).

The demands for each land‐use type in 2040 were simulated using a Markov chain based on the patch number of each land‐use type from 2000 to 2020 (Lin and Fu 2023). The transition matrix was set according to the land use between 2000 and 2020, with changed land‐use types set as 1, and unchanged set as 0. The change weight of each land‐use type was identified according to the formula below (Wang et al. 2019).

(1) $$W_{i}=TA_{i}-\frac{{\mathrm{TA}}_{\min}}{\;{\mathrm{TA}}_{\max}}-{\mathrm{TA}}_{\min}$$

*W*_*i*_ is the weight of Class *i* land‐use type, *TA*_*i*_ is the expansion area of Class _*i*_ land use, *TA*_min_ is the minimum expansion area of each land‐use type, and *TA*_max_ is the maximum expansion area of each land‐use type.

A 3 × 3 Moore neighborhood was adopted to quantify the PLUS's neighborhood effects. 0.1 was set to generate the threshold of new land use patches, and 0.9 was defined as the decay factor of the decreasing threshold. 500 was set as the step size of the PLUS to approximate the land use demand. All the parameters are set using a trial‐and‐error method (Liang et al. 2021).

Validation of the simulation results according to the Figure of Merit (FoM). A Kappa coefficient with a value close to 1 indicates higher simulation accuracy. When the coefficient reaches 0.8 or above, the simulation accuracy can be considered satisfactory (Lin and Fu 2023; Lin et al. 2020).

### 2.4 Suitable Habitat

According to CLCD, five land‐use types, cropland, forest, shrub, grassland, and barren, meet the survival requirements of *C. barometz* . The habitat at each suit level was extracted by masking the SDAs at each suit level (Figure S1).

## 3 Results

### 3.1 Suitable Distribution Area Change

To reduce the autocorrelation, 6 variables (BIO_9, BIO_8, SOM, BIO_13, BIO_3, BIO_11) with a percentage contribution smaller than 1% were removed directly, and Pearson analysis was used to examine the correlations of the remaining 19 variables. The remaining variables with an absolute correlation coefficient lower than 0.8 were kept. The variables with the highest percentage contribution among those with an absolute correlation coefficient higher than 0.8 were also kept (Figure S2). Finally, 10 variables (ALT, ASP, BD, BIO_12, BIO_18, BIO_19, BIO_2, BIO_7, PH, SLP) were chosen for the second run as the former setting in 2020 and under multiple SSPs in 2040.

The Cloglog values illustrated excellent performance for outputs of *C. barometz* with all average test AUCs higher than 0.9 (Figure S3). Altitude and BIO_18 (Precipitation of Warmest Quarter) are key environmental variables with high percentage contributions under 2020 and 2040 under three SSPs (Table S3).

**FIGURE 2:**
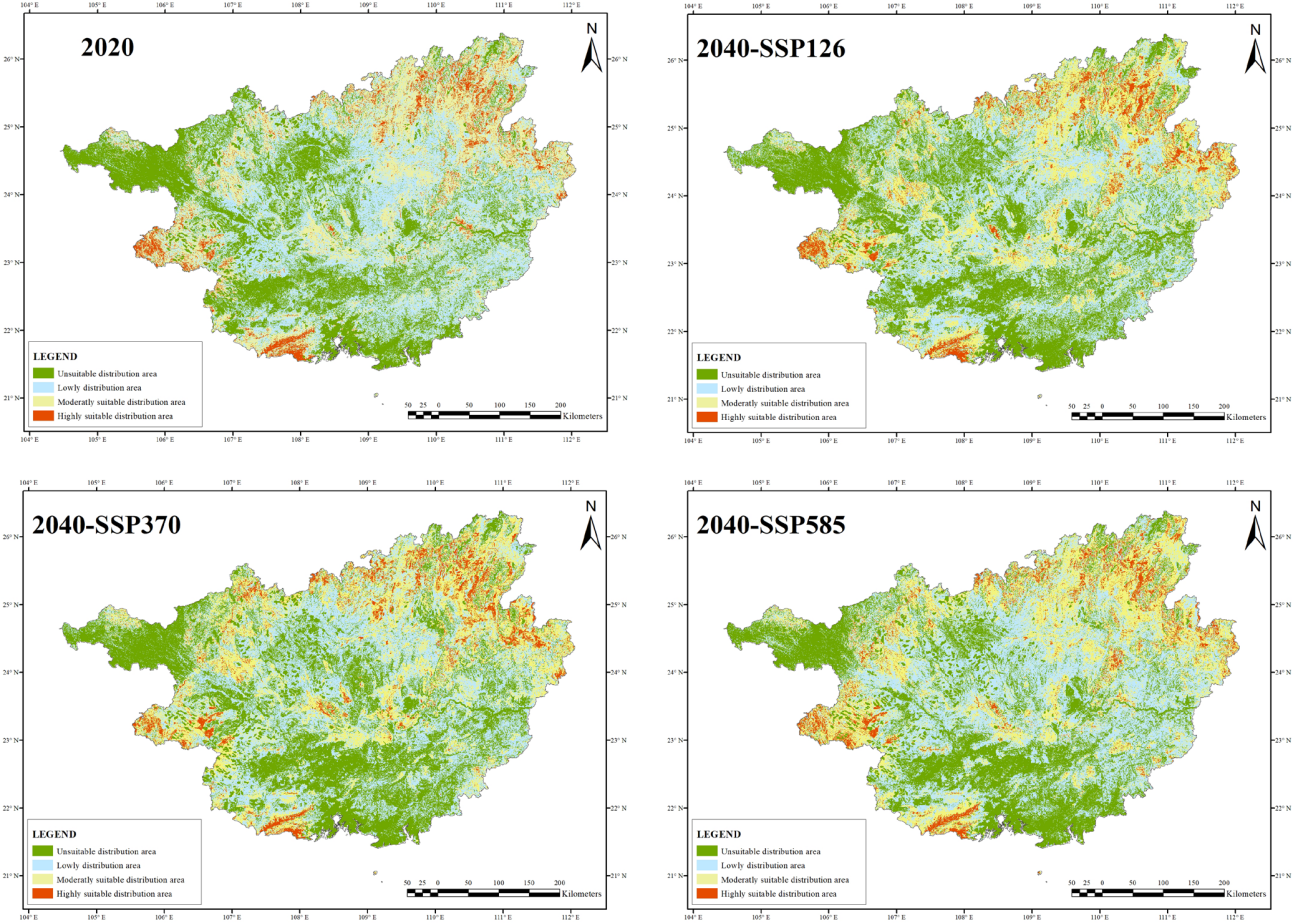
The SDA of *Cibotium barometz* in Guangxi for 2020 and 2040 under three SSPs.

**FIGURE 3:**
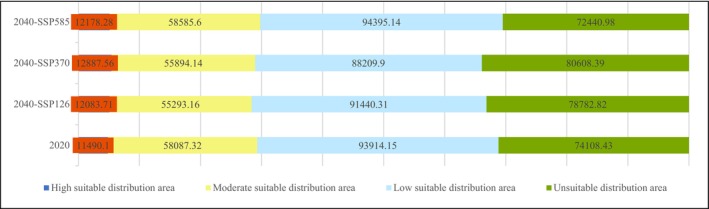
Area of *C. barometz* for 2020 and 2040 under three SSPs.

Using Manual Interval with breakpoint values of 0.8, 0.6, and 0.4, *C. barometz* 's SDAs for the present and 2040 under SSP126, SSP370, and SSP585 are 163491.57km^2^, 158817.18km^2^, 156991.61km^2^, and 165159.02km^2^, accounting for 68.81%, 66.84%, 66.07%, and 69.51% of the total area of Guangxi (Figure 2). By 2040, the SDA of *C. barometz* is projected to decrease by 2.86% (SSP126), 3.98% (SSP370), and increase by 1.02% (SSP585). The SDAs for each suitability level vary to different extents (Figure 3).

**FIGURE 4:**
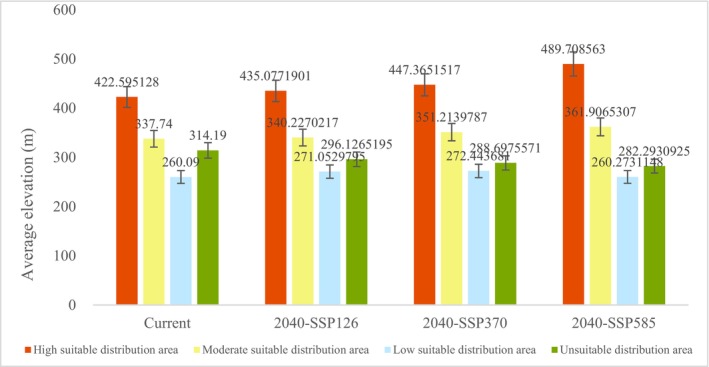
The average elevation of *Cibotium barometz* for 2020 and 2040 under three SSPs.

By 2040, the average elevation of each suit level SDA is expected to increase, except for the low SDA under SSP585. On the other hand, the unSDAs under three SSPs are expected to experience a decrease in average elevation (Figure 4).

**FIGURE 5:**
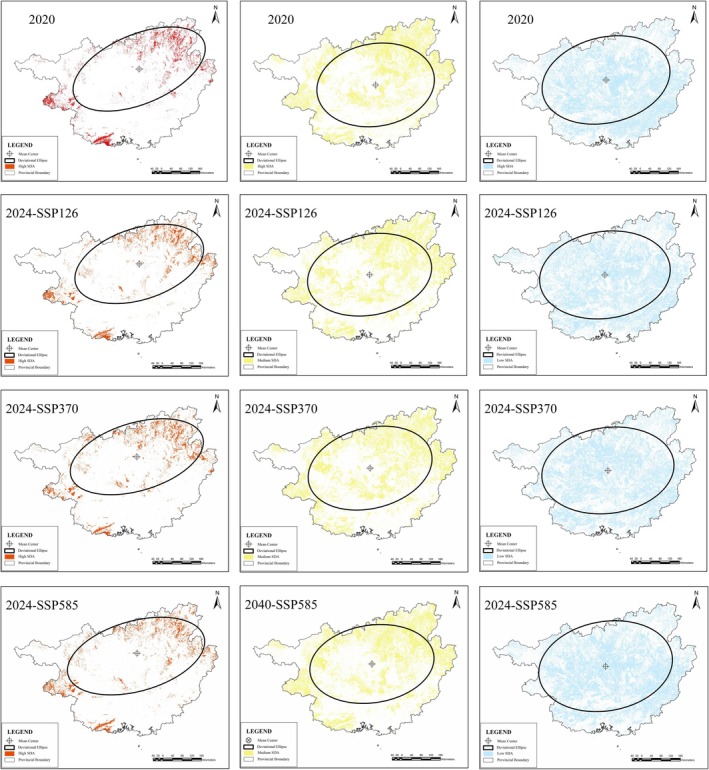
Mean center and shift tendency of *Cibotium barometz* for 2020 and 2040 under three SSPs.

In both 2020 and 2040, the elevation of the mean center of the high SDA is higher than that of the moderate and low SDAs. According to the Standard Deviational Ellipse, the high SDAs are observed to shift towards the northeast, both in 2020 and in 2040 across all three SSPs, with the trend of high SDA being much stronger than that of moderate and low SDAs (Zhang et al. 2022) (Figure 5).

### 3.2 Land Use Change

The overall accuracy of the simulation result was 90.21%, the Kappa coefficient was 0.881, and the FoM value was 0.259; the accuracy is satisfactory (Liang et al. 2021). For the most important contribution of land‐use type conversion, the drivers that have the greatest impact on the change of cropland, forest, shrubs, grassland, barren, and impervious surfaces are population (0.12), elevation (0.21), temperature (0.23), distance to water (0.23), and GDP (0.34).

**FIGURE 6:**
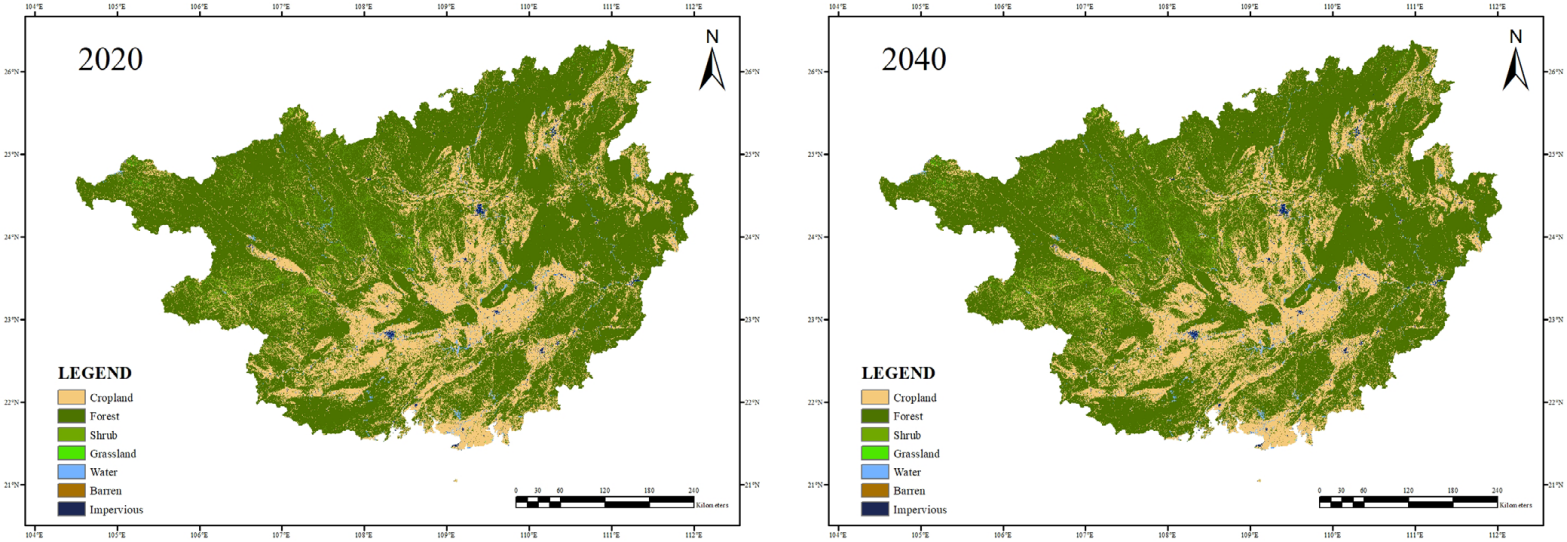
LULC of Guangxi in 2020 and 2040.

The results indicate that the areas of all land‐use types have changed to varying extents between 2020 and 2040 (Figure 6, Table S4). The areas of cropland, forest, shrub, grassland, and barren that meet *C. barometz* ’ survival requirement are expected to decrease by 0.47%, 0.26%, 16.07%, 13.71%, and 7.69% respectively, yet, the areas of water and impervious that do not meet *C. barometz* ’ survival requirement are expected to increase by 2.36% and 47.76% (Table 1, Table S5).

**TABLE 1:** Areas of each land‐use type in Guangxi in 2020 and 2040 (Unit: Km^2^).

Time	Cropland	Forest	Shrub	Grassland	Water	Barren	Impervious
2020	58596.90	168875.85	4623.64	139.12	2321.23	3.31	3039.96
2040	58319.75	168439.35	3880.51	120.04	2375.91	3.05	4461.39

### 3.3 Suitable Habitat

**FIGURE 7:**
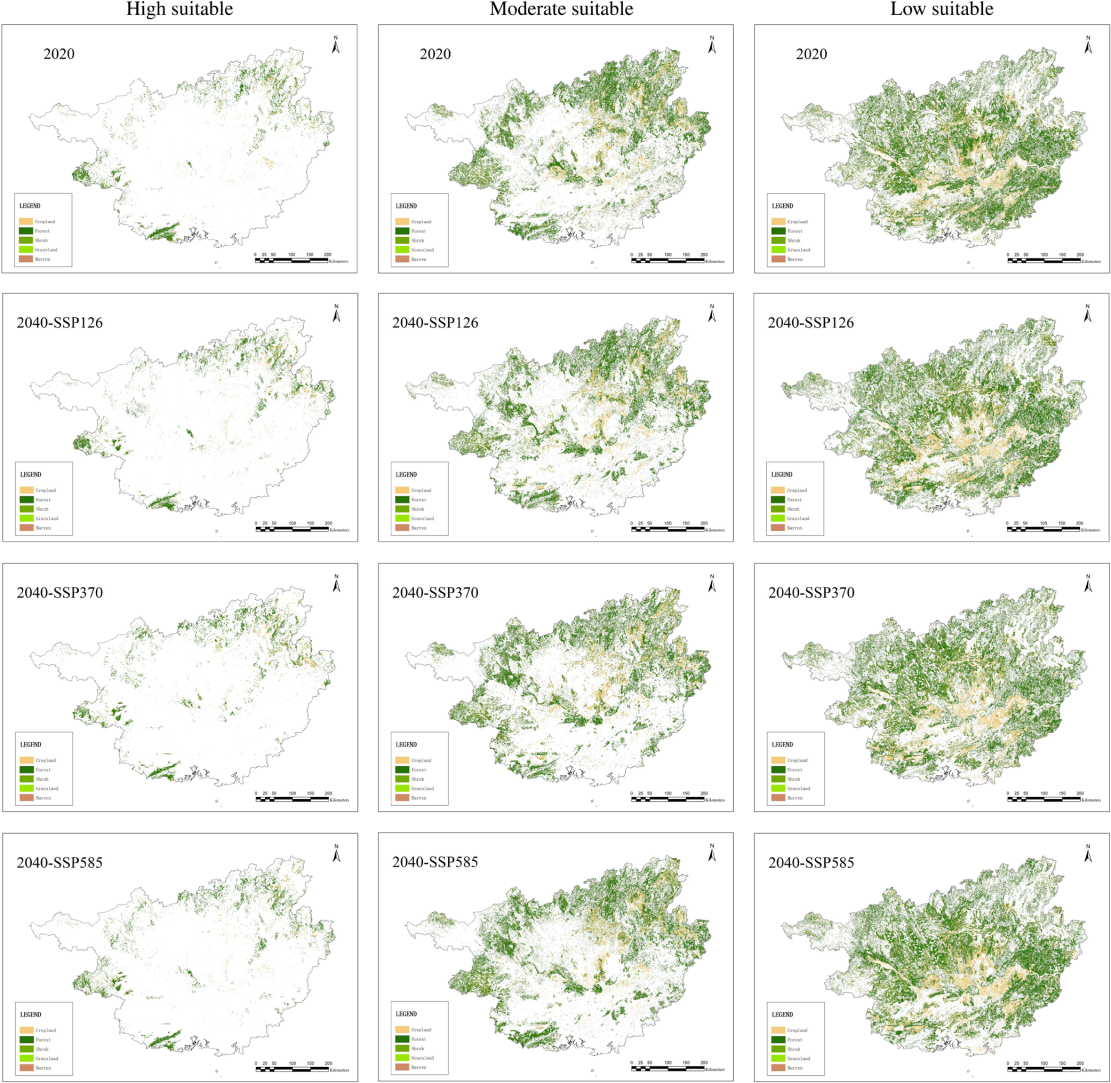
Suitable habitat of *Cibotium barometz* for 2020 and 2040 under three SSPs.

The total area of 2020 suitable habitat for *C. barometz* in Guangxi is 160161.51km^2^, including 11243.25 km^2^ of high suitable habitat, 56788.24 km^2^ of moderate suitable habitat, and 92130.02 km^2^ of low suitable habitat; including 39177.99 km^2^ of cropland, 118275.54 km^2^ of forest, 2627.64 km^2^ of shrub, 78.3 km^2^ of grassland, and 2.03 km^2^ of barren. By 2040, the 2020 suitable habitat for *C. barometz* is expected to decrease by 815.55 km^2^ (0.51%) due to land use change (Figure 7, Table S4).

The suitable habitat of *C. barometz* is expected to decrease by 3674.28 km^2^ (3.99%) and 5455.11 km^2^ (5.92%) under SSP126 and SSP370 and increase by 2695.01 km^2^ (2.93%) under SSP585. Thus, suitable habitat for *C. barometz* will experience a decrease under SSP126 and SSP370 and an increase under SSP585.

Among them, the area of high suitable habitat will increase by varying amounts across three SSPs, with increases of 679.28 km^2^ (6.04%) under SSP126, 1454.15 km^2^ (12.93%) under SSP370, and799.53 km^2^ (7.11%) under SSP585. Meanwhile, the area of medium suitable habitat will decrease by 2382.32 km^2^ (4.2%) and 1687.53 km^2^ (2.97%) under SSP126 and SSP370, respectively, and will increase by 942.91 km^2^ (1.66%) under SSP585. The area of low suitable habitat will decrease by 1971.24 km^2^ (2.14%) and 5221.73 km^2^ (5.67%) under SSP126 and SSP370, and will increase by 952.57 km^2^ (1.03%) under SSP585.

## 4 Discussion

The climate is gradually becoming warmer (IPCC 2014). Population growth and the expansion of the global economy are intensifying the exploitation of agroecology and increasing the volatility of its uses to adapt to yield requirements (Kirkby 2021). Climate and land use changes impact species' spatial distribution patterns, habitats, and survival ability (Saupe et al. 2019; Oliver and Morecroft 2014). Models that forecast the climatic space and habitat pattern of medicinal plants can play a significant role in future habitat conservation and sustainable management strategies (Porfirio et al. 2014; Hurtt et al. 2020). Therefore, we used a combination of MaxEnt and PLUS to examine the synergistic impact of climate and land use change on the habitat of *C. barometz* between 2020 and 2040. With a sample size of 123 occurrences for Maxent simulation, the accuracy and stability of the simulation were ensured (Chen et al. 2012). The AUC values of MaxEnt were all higher than 0.9 (Figure S3), indicating that the results were excellent. Meanwhile, the results of PLUS suggest that the PLUS model is suitable for simulating land use patterns in Guangxi (Liang et al. 2021; Lin and Fu 2023).

Land use can influence vulnerability independently and in conjunction with climate change, resulting in either an exacerbation or a diminution of the degree of endangerment (Mantyka‐Pringle et al. 2015). By 2040, the area of *C. barometz* ’ suitable habitat is predicted to decrease by 0.51% due to land use change; SDA is expected to decrease by 2.86% (SSP126), 3.98% (SSP370), and increase by 1.02% (SSP585). Under the synergistic impact of climate and land use change, the suitable habitat is expected to decrease under SSP126 and SSP370 and increase under SSP585. According to the Action Plan on Peak Carbon Emissions 2030 released by the Chinese government in 2021, the share of non‐fossil energy consumption will rise and greenhouse gas emissions are expected to drop significantly; therefore, the fossil‐fueled development scenario (SSP585) will not occur. This means that SDA and suitable habitat will decrease, and climate change will exacerbate habitat loss due to land use. Simultaneously, the high suitable habitat showed an increasing trend across three SSPs; the medium and low suitable habitat is expected to decrease under SSP126 and SSP370 while increasing under SSP 585. In general, the suitability degree of suitable habitat will increase by 2040, despite a slight decrease in the total areas of suitable habitat for *C. barometz* .

It can be demonstrated that species' futures have influenced their adaptions to climate change (Pacifici et al. 2017). According to the percentage contribution of MaxEnt, altitude is one of the key geographical variables in determining the distribution of *C. barometz* (Table S3). This indicates that *C. barometz* is sensitive to altitude. The species is suitable in elevations ranging from‐200 m to 1300 m, consistent with previous studies (Huang et al. 2022; Wei 2019; He 2013). The optimal distribution range for *C. barometz* is between 200 m and 300 m (Figure S4). In 2040, the average elevation of SDAs tends to increase except for the low SDA under SSP 585. At 2020 and under three SSPs in 2040, the mean center of high SDA is located at a higher latitude than moderate and low SDAs, simultaneously, the SDA of *C. barometz* tends to shift to the northeast, especially the high SDA (Figure 5). The topography of Guangxi is characterized by a high elevation in the northwest and a low elevation in the southeast, thus, there is a positive correlation between elevation and shift trend. This indicates that the SDA of *C. barometz* has a trend shifting to the high‐altitude area in northeast Guangxi, and the current low‐altitude SDA in southwestern Guangxi will no longer be suitable for *C. barometz* . Overall, the shifting trend of *C. barometz* 's SDA showed a northeast–southwest and upslope shift.

A multitude of factors affect the geographical distribution of plant life. Previous studies have demonstrated that climate significantly drives regional‐scale plant distribution (Zhang et al. 2024). According to the percentage contribution of MaxEnt, BIO_18 (Precipitation of Warmest Quarter) is another key bioclimatic variable in determining the distribution of *C. barometz* (Table S3). *C. barometz* prefers warm and humid environments (Qin 1959). Currently, the optimal BIO_18 for *C. barometz* is 1500 mm or more; in 2040, the optimal BIO_18 for *C. barometz* is 1700 mm or more (Figure S5). By 2100, precipitation is expected to increase continually in South China and Southwest China during the upcoming summer (Lu et al. 2022). It can be concluded that future precipitation in Guangxi is suitable for the habitat of *C. barometz* .

**FIGURE 8:**
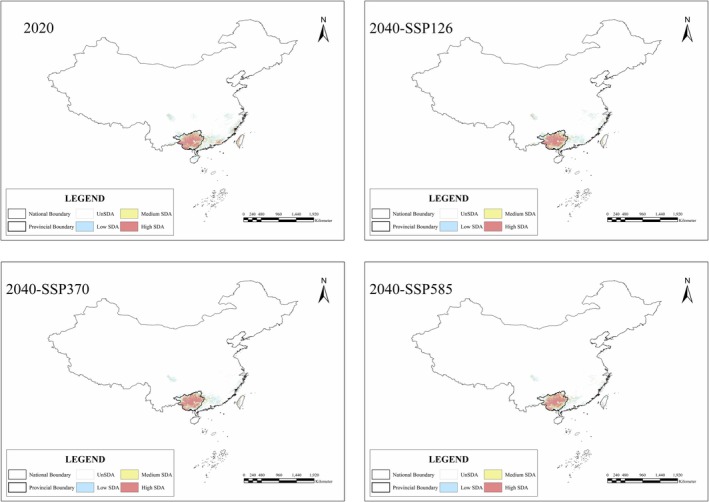
The suitable distribution area of *Cibotium barometz* for 2020 and 2040 in China under three SSPs.

Furthermore, we simulated the SDA of *C. barometz* in China using the same method as in Guangxi. Our findings indicate that in 2020, *C. barometz* 's highly suitable distribution area in Guangxi constituted 81.98% of China's total highly suitable distribution area (Figure 8). By 2040, this figure had increased to 92% (SSP126), 93.13% (SSP370), and 93.81% (SSP585), respectively. Guangxi will remain the main highly suitable distribution area for *C. barometz* in the future, thus maintaining its designation as the most significant production area.

As the main production area, the sustainable utilization of *C. barometz* in Guangxi requires a scientific strategy that takes into account the synergistic impact of climate and land‐use change on suitable habitats. To address the trend of habitat change, we propose an adaptive response to wild resource conservation based on the protected area system in southwestern Guangxi in parallel with artificial cultivation in northeastern Guangxi. Southwest Guangxi, with low altitude, is a biodiversity hotspot with a well‐developed protected areas system (Lin et al. 2022). The adaptive management aims to improve the conservation of wild resources and habitats in southwestern Guangxi, which is experiencing a gradual loss of SDA, based on the protected area system because protected areas serve to mitigate the impacts of climate change by offering a thermal buffer effect (Xu et al. 2022) and attenuate anthropogenic climate change, which includes land‐use change (Pacifici et al. 2015; Pimm et al. 2014). On the other hand, artificial cultivation should be carried out in northeastern Guangxi to meet the market demand. *C. barometz* is a perennial with a long growth cycle; the long‐term environmental suitability and stability are essential for the growth of *C. barometz* . Therefore, the priority area for artificial cultivation should be selected based on the long‐term stable and potentially suitable habitats in 2040, or those that will become suitable in northeastern Guangxi.

## 5 Conclusions

*C. barometz* is a highly demanded medicinal plant listed as a national key protected wild plant.

As the main production area, to achieve sustainable utilization of *C. barometz* , We evaluated the synergistic impact of climate and land use change in the present and 2040 under three SSPs (SSP126, SSP370, and SSP585) on Guangxi's habitat of *C. barometz* using MaxEnt and PLUS models, and propose the adaptive management strategy. We observed the following: (1) Elevation and BIO_18 are key factors for the shift of SDA. (2) The average elevation of SDA will increase; meanwhile, the SDAs show a southwest‐northeast and upland shift trend in Guangxi, particularly the high SDA. (3) Compared to the present, the areas of cropland, forest, shrub, grassland, and barren that meet *C. barometz* ’ survival requirement are expected to decrease, while the areas of water and impervious surfaces that do not meet *C. barometz* ’ survival requirement are expected to increase by 2040. (4) The area of the five land‐use types that meet the survival requirements of *C. barometz* will be diminished. The SDA and suitable habitat of *C. barometz* are projected to decrease under SSP126 and SSP370, yet increase under SSP585 according to MaxEnt simulation. For national policy on non‐fossil energy consumption, the SDA and suitable habitat are expected to decrease in 2040, which means that climate change will exacerbate the habitat loss caused by land use. (5) The suitability degree of suitable habitat will increase despite a decrease in the total area of suitable habitat by 2040. (6) We propose an adaptive management countermeasure that conserves wild resources and habitats based on the protected areas system in southwestern Guangxi in parallel with artificial cultivation in northeastern Guangxi. This study aims to offer a scientific reference for the adaptive management of *C. barometz* resources and provide insights into the conservation and sustainable utilization of endangered medicinal plants.

## Author Contributions

**Bin Feng:** conceptualization (equal), data curation (equal), formal analysis (equal), investigation (equal), methodology (equal), software (equal), visualization (equal), writing – original draft (equal), writing – review and editing (equal). **Yunyun Zhang:** data curation (equal), formal analysis (equal), investigation (equal), resources (equal), writing – review and editing (equal). **Yunfeng Huang:** methodology (equal), project administration (equal), supervision (equal), validation (equal). **Huabing Dai:** validation (equal). **Chao Yang:** software (equal), visualization (equal). **Chengling Yang:** software (equal), validation (equal). **Kedao Lai:** conceptualization (supporting), funding acquisition (lead), project administration (lead), supervision (equal), validation (equal).

## Conflicts of Interest

The authors declare no conflicts of interest.

## Supporting information


Data S1.

## Data Availability

The data supporting this study's findings are available for all interested parties on a shared drive accessible with the following link: https://datadryad.org/stash/dataset/doi:10.5061/dryad.83bk3jb3f.
