# Supplementary material for: Adaptive Management Based on the Habitat Change of Cibotium barometz Under Synergistic Impact of Climate and Land Use Change—A Case Study of Guangxi, China
Source: Ecol Evol. 2025 Mar 13;15(3):e71040. doi: 10.1002/ece3.71040 (PMC11906282; doi:10.1002/ece3.71040)
Supplement: Supplementary file 1 — Data S1. [file ECE3-15-e71040-s001.docx]

**Appendix**

**Table S1.** Environmental variables for SDA modeling

| **Category** | **Variable** | **Unit** | **Code** | **Category** | **Variable** | **Unit** | **Code** |
| --- | --- | --- | --- | --- | --- | --- | --- |
| Climate | Annual Mean Temperature | °C | BIO_1 | Climate | Precipitation of Driest Month | mm | BIO_14 |
|  | Mean Diurnal Range | °C | BIO_2 |  | Precipitation Seasonality |  | BIO_15 |
|  | Isothermality |  | BIO_3 |  | Precipitation of Wettest Quarter | mm | BIO_16 |
|  | Temperature Seasonality |  | BIO_4 |  | Precipitation of Driest Quarter | mm | BIO_17 |
|  | Max Temperature of Warmest Month | °C | BIO_5 |  | Precipitation of Warmest Quarter | mm | BIO_18 |
|  | Min Temperature of Coldest Month | °C | BIO_6 |  | Precipitation of Coldest Quarter | mm | BIO_19 |
|  | Temperature Annual Range | °C | BIO_7 | Topography | Altitude | m | ALT |
|  | Mean Temperature of Wettest Quarter | °C | BIO_8 |  | Slope | ° | SLP |
|  | Mean Temperature of Driest Quarter | °C | BIO_9 |  | Aspect | ° | ASP |
|  | Mean Temperature of Warmest Quarter | °C | BIO_10 | Soil | pH Value (H_2_O) |  | PH |
|  | Mean Temperature of Coldest Quarter | °C | BIO_11 |  | Soil Organic Matter | g/100g | SOM |
|  | Annual Precipitation | mm | BIO_12 |  | Bulk Density | g/cm^3^ | BD |
|  | Precipitation of Wettest Month | mm | BIO_13 |  |  |  |  |

**Table S2.** Environmental variables for PLUS modeling

| **Category** | **Data** | **Year** | **Category** | **Data** | **Year** |
| --- | --- | --- | --- | --- | --- |
| Land use/cover data | Land use/cover data | 2000 | Socio-economic driver | Proximity to the secondary road | 2020 |
|  |  | 2020 |  | Proximity to the tertiary road | 2020 |
| Socio-economic driver | Population | 2019 |  | Proximity to high-speed railway stations | 2020 |
|  | GDP | 2019 | Climatic and environmental driver | Soil type | 2014 |
|  | Proximity to governments | 2020 |  | Proximity to open water | 2020 |
|  | Proximity to highway | 2020 |  | Annual Mean Temperature | 2020 |
|  | Proximity to railway | 2020 |  | Annual Precipitation | 2020 |
|  | Proximity to the arterial road | 2020 |  | Altitude | 2020 |
|  | Proximity to the primary road | 2020 |  | Slope | 2020 |

**Table S3.** Percent contribution of key environmental variables

| **Environmental variable** | **2020** | **2040-SSP126** | **2040-SSP370** | **2040-SSP585** |
| --- | --- | --- | --- | --- |
| Top 1 | ALT (15.2) | ALT (16.8) | ALT (15) | ALT (15) |
| Top 2 | BIO_19 (12.9) | BIO_19 (12.8) | BIO_7 (10.2) | BIO_19 (12.9) |
| Top 3 | BIO_18 (9.5) | BIO_7 (12.3) | Ph (9.5) | BIO_18 (10.3) |
| Top 4 | BIO_7 (7.6) | Ph (10.6) | BIO_18 (9.1) | Ph (9.3) |
| Top 5 | SLP (7.3) | BIO_18 (8.9) | SLP (8.2) | SLP (7.6) |

**Table S4.** Areas change of land-use types between 2020 to 2040

| **Land use types** | **Cropland** | **Forest** | **Shrub** | **Grassland** | **Water** | **Barren** | **Impervious** |
| --- | --- | --- | --- | --- | --- | --- | --- |
| Cropland | 46354.73 | 10586.83 | 81.89 | 48.32 | 281.28 | 2.29 | 1355.56 |
| Forest | 11214.51 | 155398.02 | 1675.75 | 39.42 | 21.62 | 0.76 | 158.45 |
| Shrub | 636.32 | 2732.99 | 2834.72 | 22.13 | 0.76 | 0 | 0.51 |
| Grassland | 109.36 | 98.17 | 31.28 | 26.20 | 13.22 | 0.25 | 16.79 |
| Water | 272.38 | 59.51 | 0 | 2.29 | 1946.1 | 0 | 34.08 |
| Barren | 0.51 | 0 | 0 | 0.51 | 0.25 | 0 | 0.76 |
| Impervious | 9.16 | 0.51 | 0 | 0.25 | 57.99 | 0 | 1473.82 |
| Total | 58596.90 | 168875.85 | 4623.64 | 139.12 | 2321.23 | 3.31 | 3039.96 |

**Table S5.** Suitable habitat at each suitable level under 2020 and multi-SSP in 2040

| Suit level | Land-use type | 2020 | 2040-SSP126 | 2040-SSP 370 | 2040-SSP 585 |
| --- | --- | --- | --- | --- | --- |
| High suitable habitat | Cropland | 2229.68 | 2317.93 | 2798.61 | 1921.95 |
|  | Forest | 8866.33 | 9448.99 | 9708.66 | 9905.76 |
|  | Shrub | 146.24 | 152.85 | 178.54 | 207.78 |
|  | Grassland | 5.09 | 7.63 | 8.39 | 12.21 |
|  | Barren | 0.51 | 0 | 0 | 0 |
| Moderate suitable habitat | Cropland | 13865.37 | 12598.07 | 12409.86 | 12629.60 |
|  | Forest | 42109.10 | 40775.67 | 41492.36 | 43693.56 |
|  | Shrub | 810.03 | 997.47 | 1155.41 | 1358.87 |
|  | Grassland | 26.20 | 56.71 | 65.62 | 72.48 |
|  | Barren | 0.76 | 0.25 | 0 | 0.25 |
| Low suitable habitat | Cropland | 23098.95 | 22731.71 | 21413.02 | 22822.25 |
|  | Forest | 67348.47 | 64837.50 | 62751.77 | 67356.35 |
|  | Shrub | 1672.45 | 2515.29 | 2672.72 | 2837.27 |
|  | Grassland | 47.05 | 110.89 | 106.05 | 104.27 |
|  | Barren | 0.76 | 0.25 | 0.25 | 0.51 |
| Unsuitable habitat | Cropland | 19482.43 | 20962.10 | 21988.31 | 21236.01 |
|  | Forest | 50442.87 | 53407.30 | 54516.68 | 47513.79 |
|  | Shrub | 1937.46 | 2557.76 | 2216.71 | 1819.45 |
|  | Grassland | 66.12 | 110.63 | 105.80 | 96.90 |
|  | Barren | 2.03 | 1.02 | 1.27 | 0.76 |


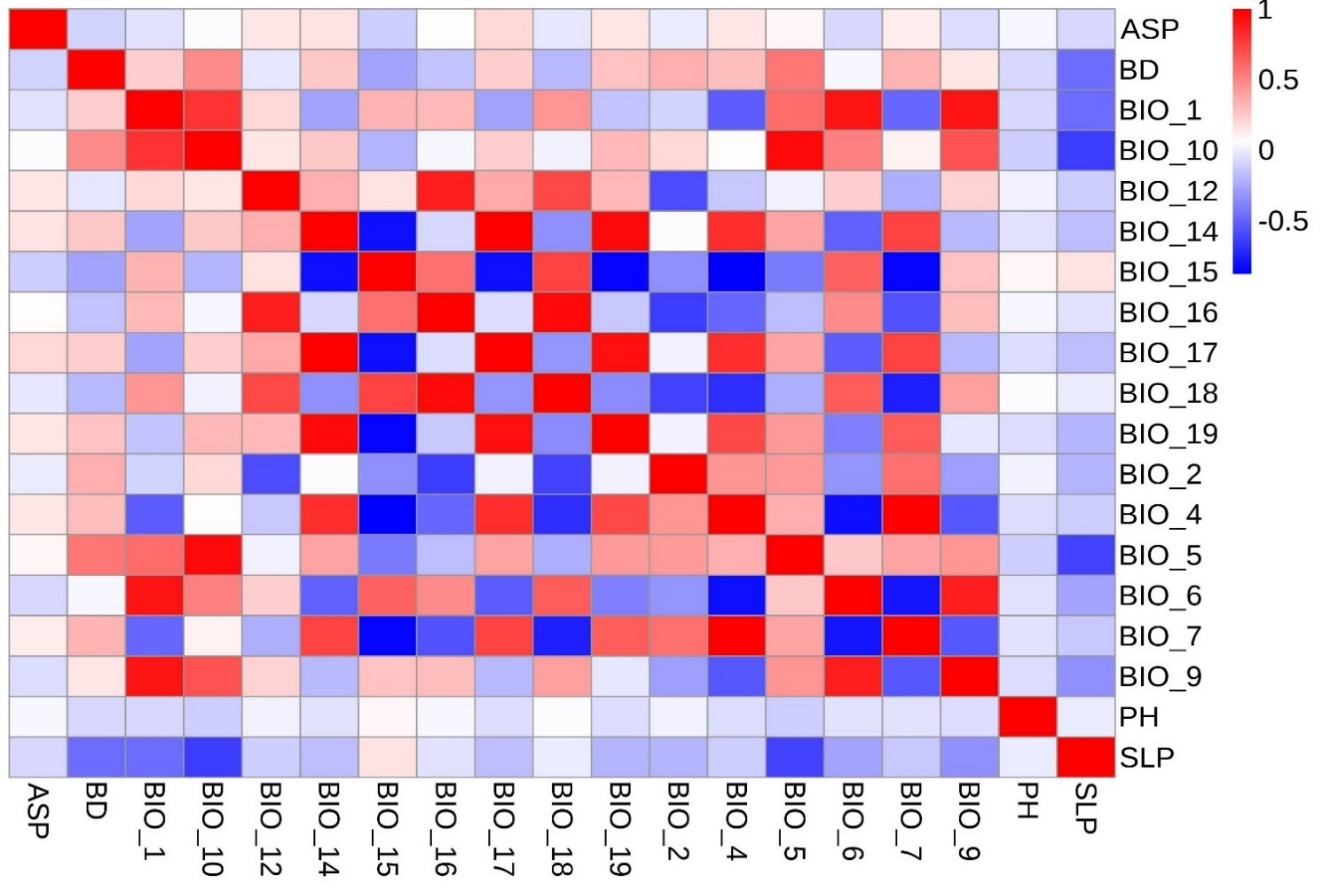

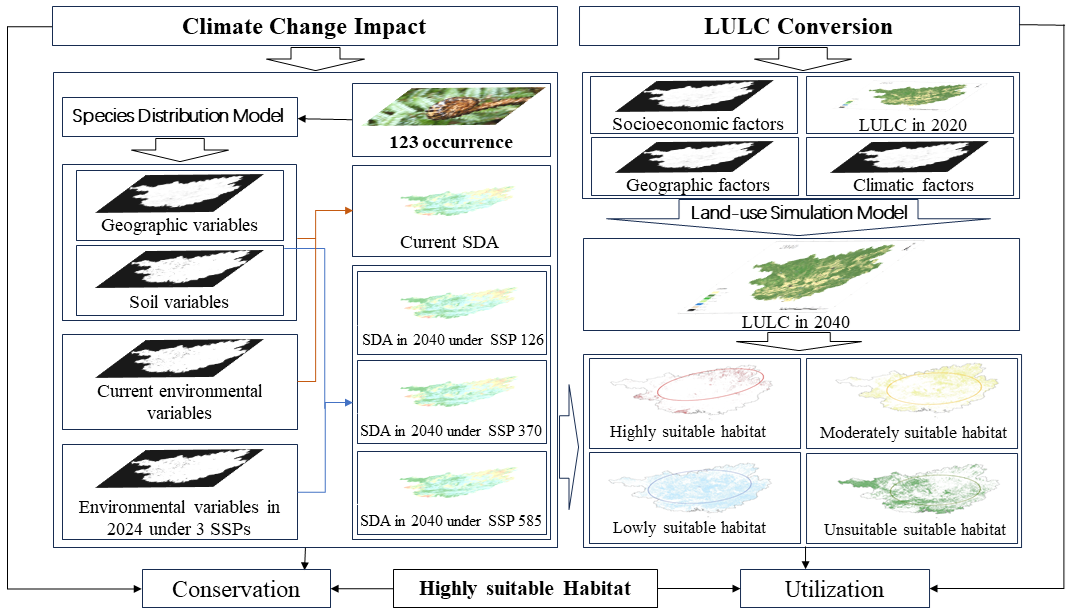
**Figure S1**. Research flowchart

**Figure S2**. CorHeatmap for Pearson correlation analysis of environmental variables.

| 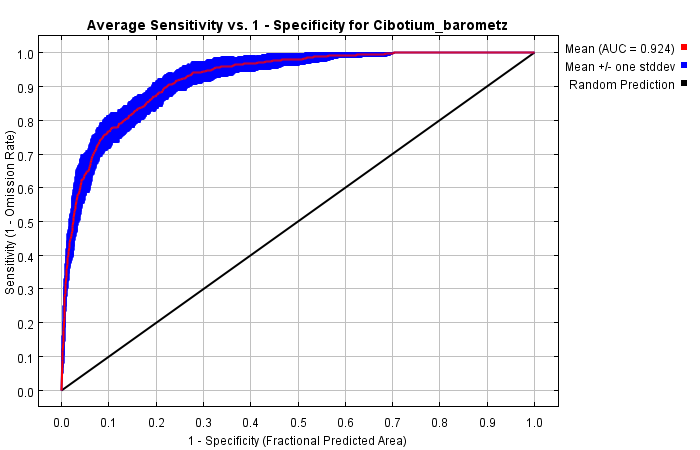a | 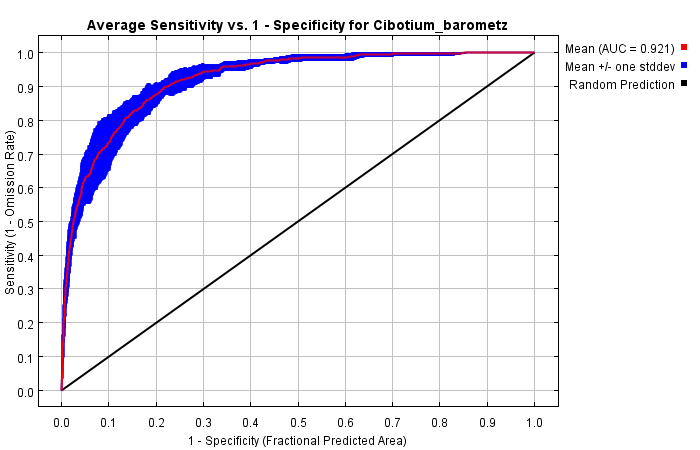b |
| --- | --- |
| 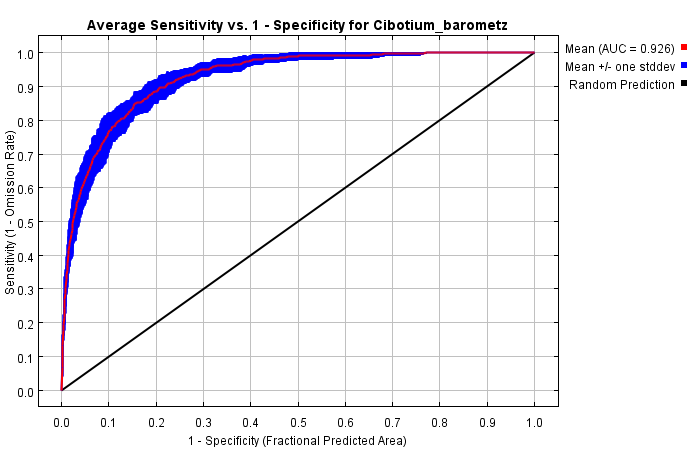c | 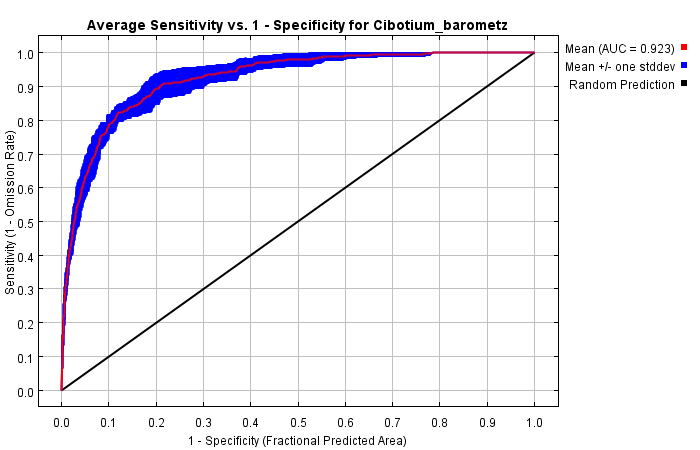d |

**Figure S3.** AUC training values for *C. barometz*: (a) 2020, (b) 2040-SSP126, (c) 2040-SSP 370, (d) 2040-SSP 585.

| 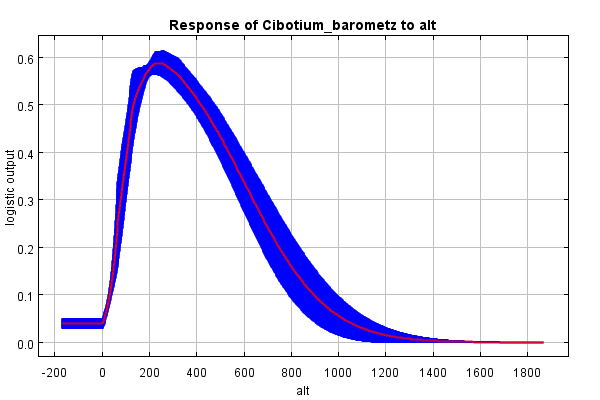a | 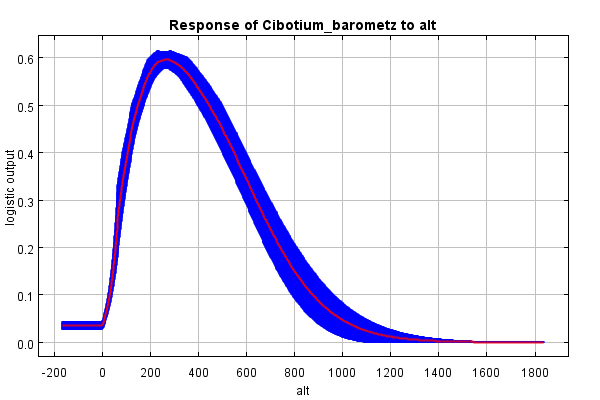b |
| --- | --- |
| 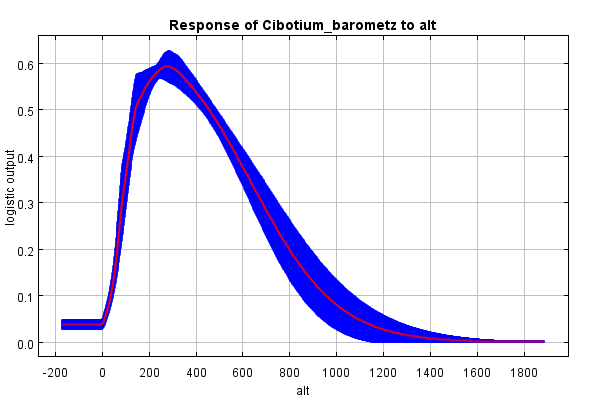c | 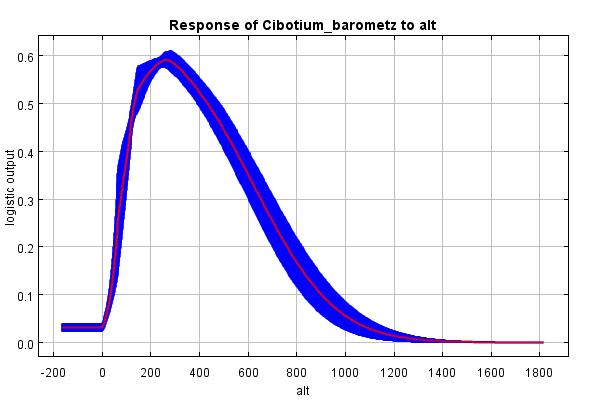d |

**Figure S4.** Response curves of altitude: (a) 2020, (b) 2040-SSP 126, (c) 2040-SSP 370, (d) 2040-SSP 585.

| 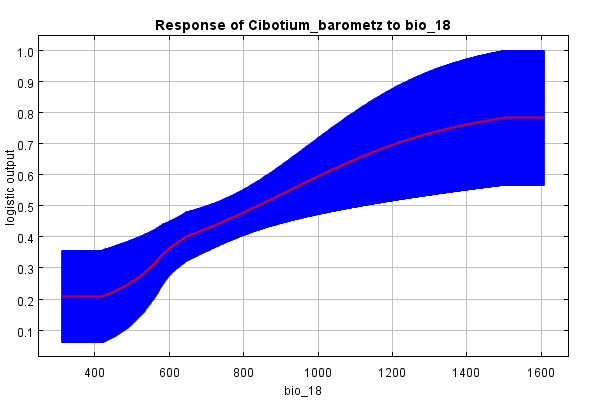a | 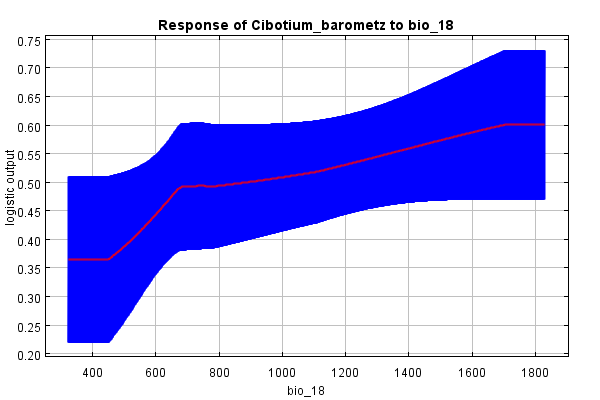b |
| --- | --- |
| 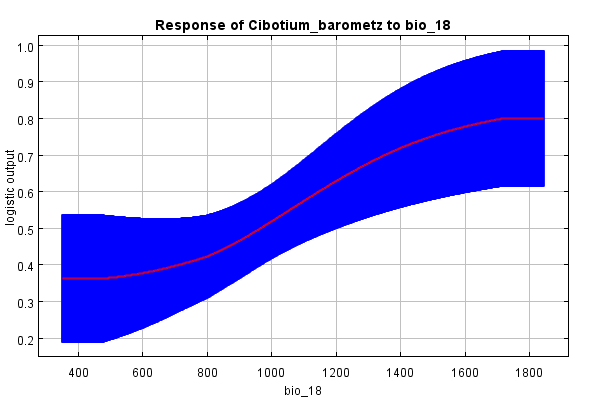c | 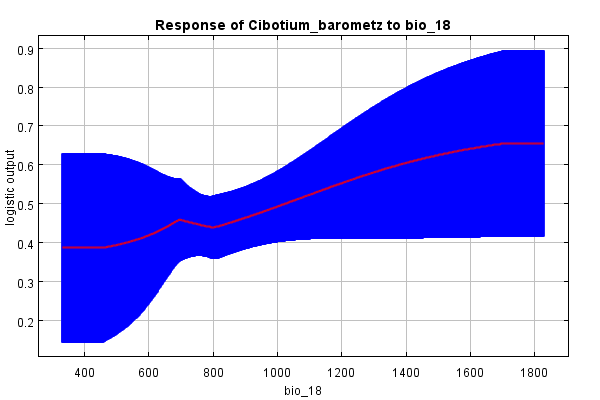d |

**Figure S5.** Response curves of BIO_18: (a) 2020, (b) 2040-SSP 126, (c) 2040-SSP 370, (d) 2040-SSP 585.
